# Morphological adaptation of the root system of silver fir responding to changes of local environmental conditions

**DOI:** 10.1371/journal.pone.0361334

**Published:** 2026-11-04

**Authors:** Aleh Marozau, Ewelina Bagińska, Justyna A. Nowakowska, Katarzyna Zabielska-Adamska, Patryk Dobrzycki, Tomasz Oszako, Konrad Wilamowski, Oleksandr Kychyliuk, Kamil Pilch, Piotr Borowik

**Affiliations:** 1 Faculty of Civil Engineering and Environmental Sciences, Institute of Forest Sciences, Białystok University of Technology, WiejskaBiałystok, Poland; 2 Faculty of Biology and Environmental Sciences, Institute of Biological Sciences, Cardinal Stefan Wyszyński University in Warsaw, Warsaw, Poland; 3 Faculty of Civil Engineering and Environmental Sciences, Institute of Civil Engineering, Białystok University of Technology, WiejskaBiałystok, Poland; 4 Forest Protection Department, Forest Research Institute, Sękocin Stary, Poland; 5 Department of Natural Forests, Forest Research Institute, Park Dyrekcyjny, Białowieża, Poland; University of Education, PAKISTAN

## Abstract

The study aimed to verify allometric changes in the root system structure of nearly 100-year-old silver fir (*Abies alba* Mill.) growing in Białowieża Forest. Traditional views of this species as possessing a simple taproot with 4–5 strong diagonal roots required supplementation with evidence of significant modification under specific conditions. It was observed that it developed extensive lateral root networks reaching 41.8–44.2 m diameter in the upper soil profile, exceeding previously reported maxima (21.2 m). This resulted from reduced competition after Norway spruce (*Picea abies* (L.) H.Karst) mortality due to drought, low groundwater level, and bark beetle infestation. The allometric shift in favor of lateral roots shows significant plasticity despite advanced age. The results confirm the climax nature of the species and show that the studied species has significant morphological adaptation. Remarkable are the significant spatial parameters (especially horizontal) of the distribution of the thin roots in the horizon of soil parent rock: depth 1.95–2.40 m, distance from the tree 6.40–10.38 m.They belong to a flattened bundle of roots growing parallel to the soil surface from the tip of the taproot and diagonal roots. Root systems were examined using ground-penetrating radar and traditional excavation methods combined with soil coring. Root identification relied on distinctive visual characteristics and anatomical analysis.

## Introduction

Silver fir (*Abies alba* Mill.) is one of Poland’s key forest-forming species, notable for its unique biological and ecological characteristics. It is considered a fundamental species for maintaining biodiversity in forest ecosystems but is sensitive to both biotic and abiotic stressors. The health of silver fir is widely used as a dendroindicator for forest ecosystem conditions, with assessments often focusing on above-ground features like crown condition [1].

Despite the ecological importance of root systems in forest dynamics, detailed knowledge of the root architecture of old-growth silver firs, particularly outside their continuous natural range, remains limited and fragmented. Against the backdrop of environmental changes in the Białowieża Forest (further Forest), manifested in prolonged droughts that caused a drop in the groundwater level by 0.5–1.5 meters [2–5] and a soil moisture deficit [2], as well as due to the subsequent invasion of bark beetles, a reduction in spruce stands occurred [3]. This allowed secondary successional species to gain a foothold [6], resulting in significant changes in the forest structure. These environmental and phytocenotic shifts are expected to provoke adaptive modifications in *A. alba* root systems, although the extent of such changes, especially in lateral root development and root plasticity overall in mature trees, is still unclear.

In conditions of climate aridization and the water factor, which is therefore at a minimum [7], root system competition and their plasticity are key determinants of resource acquisition [8]. Moreover, competition may involve many more rivals and be more pronounced than the relationships between aboveground parts, which ultimately significantly affects the overall productivity and stability of mixed stands, as well as their constituent components [9].

It is generally accepted that the root system of silver fir is taproot and consists of a skeletal vertical main root and 4–5 powerful “finger-like” roots directed diagonally (angle to the surface greater than or equal to 45° [10] to a depth of 2 m (3 m) [11].

Under the current soil moisture deficit conditions in Forest, the taproot system of silver fir should theoretically tend to penetrate deeper into the soil. Whether this is the case, and if so, how deep its maximum penetration is, remains unknown.

On the taproot and diagonal roots, a “crown” of absorption roots is formed, located parallel to the soil surface [12–14]. Information on their depth and extent is presented very fragmentarily [11].

Also poorly covered, and sometimes contradictory is the content related to the roots of *A. alba*, which are called “shallow” [11], “lateral”, “superficial”, “fine or thin” [14,15]. They are located conditionally horizontally, i.e., at an angle to the soil surface of less than 45° [10]. The article uses the term “lateral,” “thin lateral.”

Thus, in the already mentioned article by Potepa et al. [14] it is stated that they are insignificant in silver fir, while the radius of horizontal distribution in the Silesian Beskids and the Beskid Żywiecki Western Carpathians in a tree stand aged 40–60 years is only 2.5 m. At the same time, according to Brunner et al. [15], in a 100-year-old silver fir stand, roots of one of the genotypes were found at a distance significantly greater, but not more than 8.2 m from the trunk. That is, in this case, the diameter of the sphere of distribution of lateral roots slightly exceeds 16 m. Gulder [13] report that in silver fir trees it reaches 20 m and is limited by the projection of the crowns, although, as the author notes, without providing specific data, very often the zone of coverage of lateral roots is significantly larger. Kutschera and Lichtenegger [11] indicate approximately the same value – 21.2 m, with the degree of root spread increasing with increasing crown size. According to Brunner et al. [15], namely the development of the finest roots is the factor that determines the outcome of competition, but very little is known about the degree of overlap between these roots in different trees. In any case, as the authors note, it is significant in both horizontal and vertical directions between neighboring trees.

Understanding the allometric and morphological responses of *A. alba* roots to changing environments is vital for predicting the resilience of forests beyond the species’ natural distribution. This study addresses the existing knowledge gap by examining allometric adaptations in the root systems of old-growth silver firs in the Forest. Specifically, it aims to determine whether modifications in the root system correlate with environmental stressors such as groundwater shortages and structural changes in forest stands following spruce dieback.

Although silver fir has been extensively studied with respect to its regeneration, productivity, crown condition, and ecological requirements, relatively little attention has been paid to the plasticity of root system architecture in mature trees exposed to rapidly changing environmental conditions. Existing studies generally describe the root system of *Abies alba* as a relatively stable taproot system with several diagonal skeletal roots, whereas quantitative information on possible large-scale modifications of lateral root development remains scarce. In particular, the response of old-growth silver firs to long-term groundwater decline, drought stress, and changes in interspecific competition following stand restructuring has not been sufficiently documented. Therefore, the novelty of the present study lies in the investigation of root system morphology of nearly 100-year-old silver fir trees growing outside the species’ typical mountain distribution range and subjected to substantial environmental changes. The study combines ground-penetrating radar, excavation techniques, soil coring, and anatomical verification of roots to evaluate the extent of belowground morphological adaptation. This approach provides new insights into the capacity of mature silver fir trees to modify their rooting patterns under changing ecological conditions.

We propose four working hypotheses regarding possible adaptive responses: (I) **Vertical Compensation**: Increased development of the taproot and diagonal roots to access deeper soil water reserves. (II) **Horizontal Expansion**: Enhanced lateral root growth in the upper organic soil layers to compensate for reduced ground water and nutrient availability. (III) **Combined Strategy**: simultaneous development of both vertical and horizontal root systems. (IV) **Reduced Plasticity**: Limited adaptive response due to age-related decline.

## Materials and methods

### Observation site

#### macrolocation and Environmental Conditions.

The study site is located in Białowieża Forest, which is the largest remaining forestcomplex in Eastern Europe that retains its natural character. It straddles the border between Poland and Belarus, situated in the easternmost part of the Central European Lowlands and occupies an area of about 62,000 hectares [16]. The Białowieża Forest lies on a smooth, elevated plain within the Central European Lowland [17]. Geographic coordinates of subcompartment 498 Ci, where was provided our research, are N 52°42’, E 23°49’ [18]. In accordance with the official Białowieża Forest world heritage site management plan, scientific research and monitoring within subcompartment 498 Ci do not require explicit administrative permission or entry permits.

The geomorphological, climatic, geobotanical, and phytocoenotic conditions in the studied Forest differ markedly from those in the natural range of *A. alba*, which is typically for the mountainous regions of Central, Eastern, and Southern Europe [19–22]. For example, annual precipitation, a critical climatic factor for silver fir, reaches 1,200 mm on the Swiss Plateau [15], whereas here it is nearly half that, at 637 mm [23,24].

The Forest is currently undergoing rapid climatic changes, marked by warming trends and frequent, prolonged droughts. These changes have altered the climax species composition of tree stands [25].

Between 2012 and 2016, a mixed stand dominated by *P. abies* experienced rapid collapse due to the combined effects of drought and infestation by the bark beetle *Ips typographus* L. (Fig 1). In contrast, scattered silver fir individuals were largely preserved. The breakdown of the spruce canopy created extensive open areas, triggering diverse secondary successions and further altering ecological and phytocoenotic conditions.

**Fig 1:**
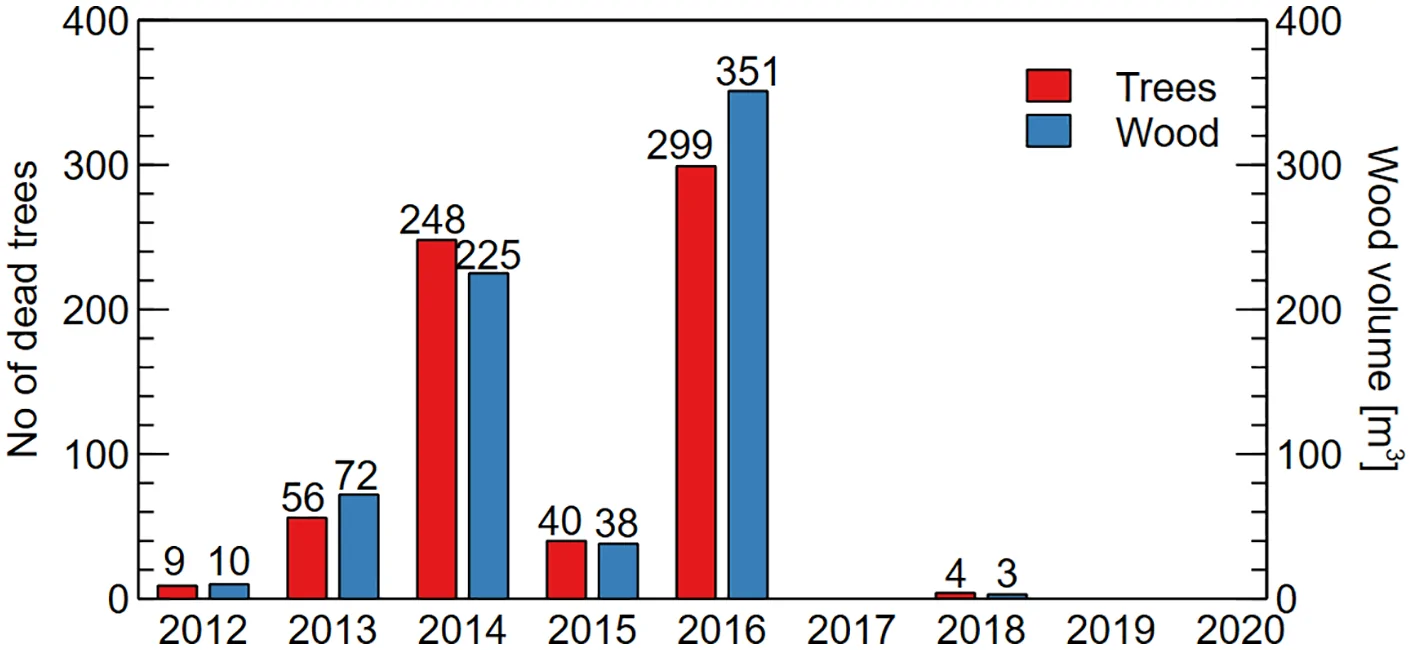
The number of dead trees of Norway spruce and stocks of its dead wood in the studied stand with the participation of silver fir (the data from Białowieża forest enterprise).

#### Microlocation and site conditions.

The studied stand is located within the Białowieża Forest Enterprise, specifically in the Grudki Forest District. According to the most recent forest inventory, the stand is classified as a mesotrophic deciduous forest.

The soil at the site is rusty proper. Its profile is distinctly differentiated into genetic horizons (Fig 2). Notably, the eluvial leaching horizon, typically found beneath the humus layer in belic-rusty soils and characterized by its lighter coloration, is absent [26]. This fact suggests that the volume of precipitation infiltrating the soil profile is insufficient to leach soluble substances into deeper layers. In other words, this may serve as an indicator of limited rainfall in the area.

**Fig 2:**
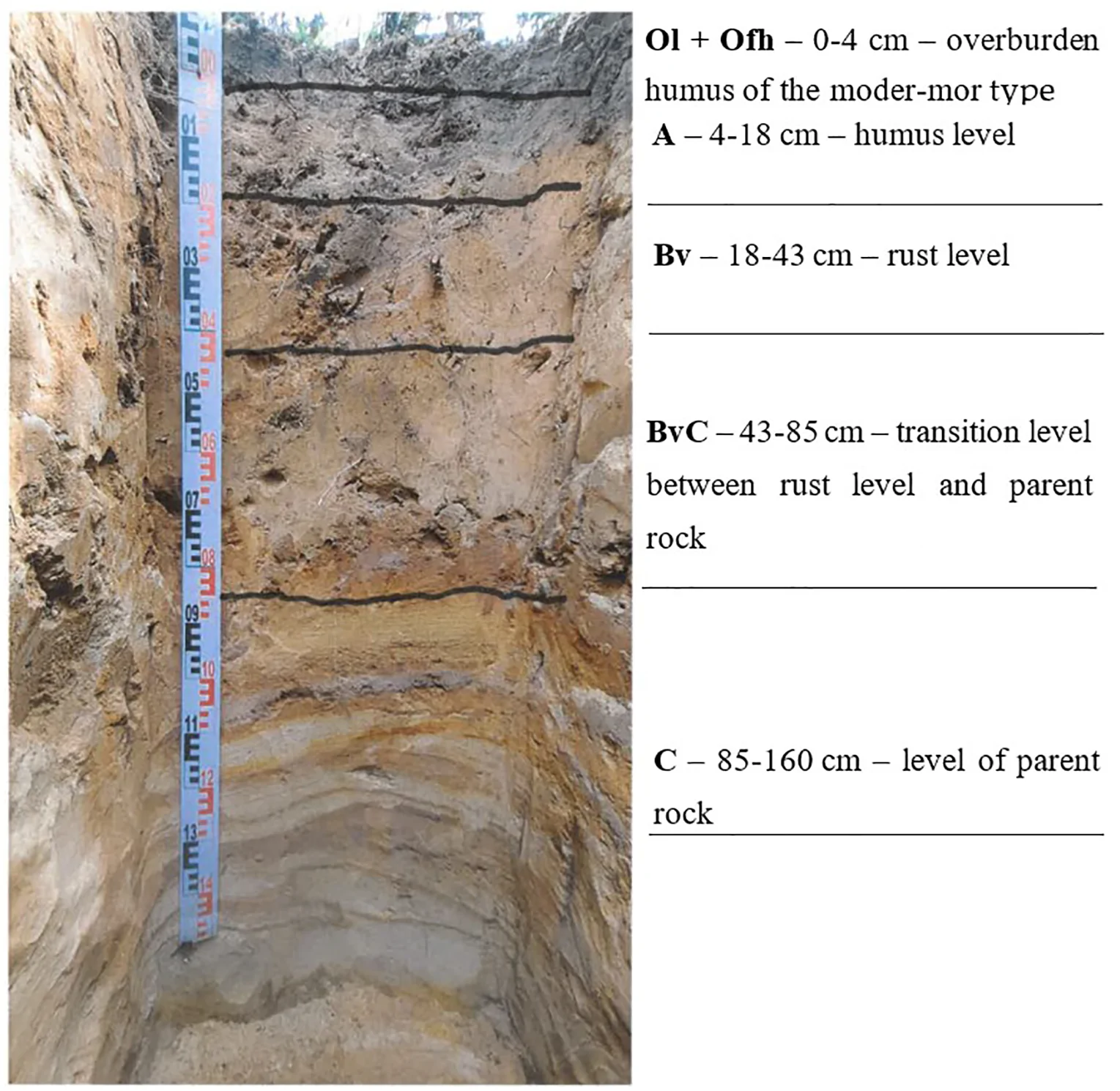
Soil profile with horizons (photo A. Marozau).

Conversely, the lack of leaching may also point to favorable conditions for humus accumulation. This interpretation is supported by the analysis presented below, which examines the ecological composition of the living ground cover (Fig 3).

**Fig 3:**
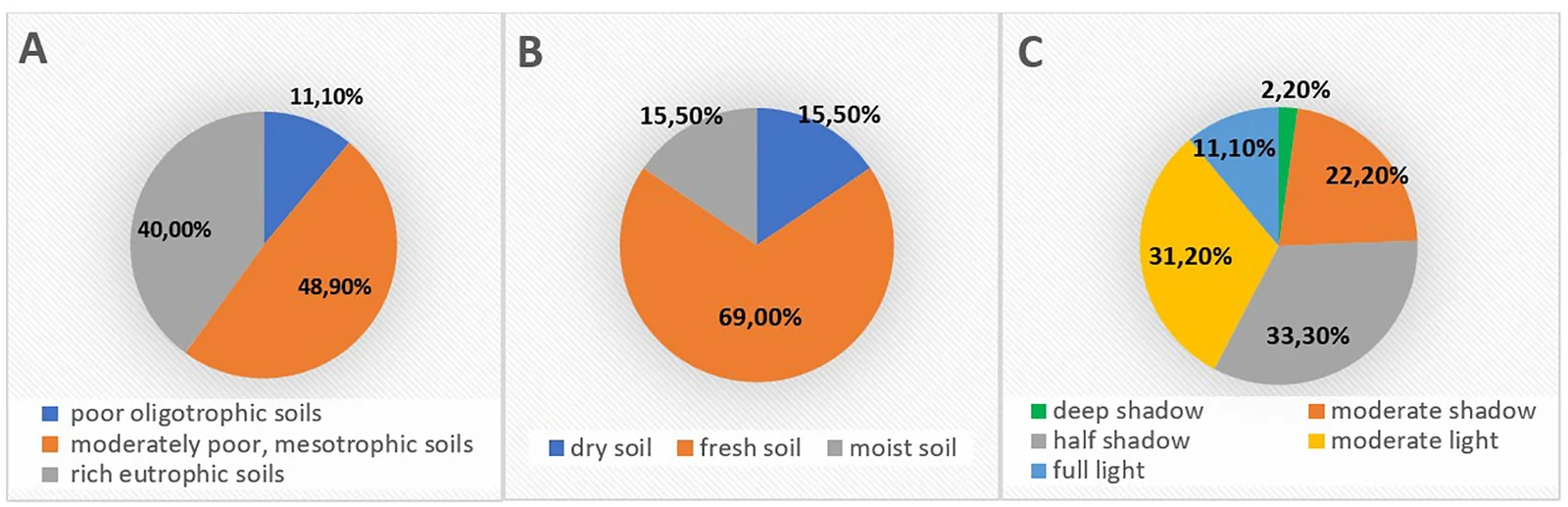
The structure of ecological numbers of plants of the living ground cover characterizing. (A) the trophism of the soil, (B) its moisture and (C) the lighting conditions.

That reveals a dominance of species associated with mesotrophic and eutrophic soils (Fig 3A) Analysis of ecological plant groups indicates also that site moisture is moderate, with no clear influence from groundwater (Fig 3B). This underscores the importance of atmospheric inputs, including horizontal precipitation, for silver fir growth under these conditions. Light conditions vary considerably across the relatively small area occupied by biogroup No. 1–5. This mosaic is shaped by the interplay of dense silver fir crowns, open gaps left by spruce removal, and shading from the closed canopy of secondary successional communities. As a result, a diverse array of plant species adapted to varying light regimes is present (Fig 3C).

Results of detailed analysis of soil conditions show, that the genetic horizons of the soil are characterized by very strong acidification of all layers. The pH of the humus horizon A is 3.44 (pH in H_2_O) and 2.94 (pH in KCl). In the rust horizon Bv, these values increase to 4.02 (pH in H_2_O) and 3.24 (pH in KCl), respectively, while in the bedrock horizon C, they are 4.36 (pH in H_2_O) and 3.81 (pH in KCl), respectively (Table 1).

**Table 1:** Chemical composition and properties of soil.

Horizon	Depth	CaCO_3_	pH	pH	Hh	Ca	Mg	K
	[cm]	[%]	H_2_0	KCl	[cmol/kg]	[cmol/kg]	[cmol/kg]	[cmol/kg]
Ofh	0-4	<1	4.25	3.37	27.30	14.132	2.708	1.181
A	4-18	<1	3.44	2.94	9.86	0.223	0.064	0.058
Bv	18-43	<1	4.02	3.24	4.37	0.542	0.269	0.065
BvC	43-85	<1	4.35	3.79	2.18	0.158	0.048	0.037
C	85-160	<1	4.36	3.81	0.96	0.742	0.226	0.061
**Horizon**	**Depth**	**Na**	**Cations**	**Sorption**	**Saturation**	**Cog**	**Nog**	**C/N**
	**[cm]**	**[cmol/kg]**	**[cmol/kg]**	**[cmol/kg]**	**[%]**	**[%]**	**[%]**	
Ofh	0-4	0.075	18.096	45.40	39.86	19.00	0.93	20.3
A	4-18	0.041	0.386	10.25	3.76	2.19	0.09	25.3
Bv	18-43	0.037	0.912	5.28	17.27	0.65	0.04	18.7
BvC	43-85	0.039	0.281	2.46	11.42	0.18	0.01	13.0
C	85-160	0.022	1.050	2.01	52.24	0.02	0.00	5.3

The overlying humus horizon reaches a thickness of 4 cm. It occurs in the fresh moder subtype. Moder humus is characterized by a slower rate of decomposition of plant debris. Ectohumus forms a layer several centimeters thick, with two distinct sub-horizons: a raw humus layer (Ol) and a detritus layer (Ofh).

The humus horizon A, 14 cm thick, is composed of slightly clayey sands. It gradually transitions into the rusty horizon Bv *sideric*, 25 cm thick. It is quite well rooted, with visible traces of humus erosion in the form of root drips. The parent rock horizons exhibits a grain size of loose sands. It is a well-ventilated horizon with a flushing type of water management.

Soil fertility is indicated by the degree of saturation of the soil’s sorption complex with base cations, which is the percentage of the total retained base cations (Ca++, Mg++, K + , Na+) in the soil’s sorption complex. The degree of saturation of individual soil horizons usually increases deeper into the soil outcrop.

The degree of base saturation in the analyzed soil is low, reaching 3.76% in the humus horizon A, and 17.27% and 11.42% in the rust horizon Bv and the transition horizon BvC. Only in the parent rock horizon C does the degree of saturation increase significantly, reaching 52.24%.

The carbon content in the A humus horizon is 2.19%. In the lower horizons it is significantly lower and does not exceed 1%. The nitrogen content in the A humus horizon is 0.09%. Even less of it was noted in the deeper layers (Table 1). The carbon-to-nitrogen (C:N) ratio is an indicator of the availability of nitrogen from plant residues in the humus-mineral horizons to plants. During humus decomposition, carbon is converted into volatile carbon dioxide. Nitrogen remains in the soil in the form of ammonium or nitrate compounds. This reduces the carbon content, increases the nitrogen content, and decreases the C:N ratio. The lower the ratio, the more efficient the humus decomposition and the more available the nitrogen is to plants. The C:N values of fertile soils of forest habitats are close to 10. In poor soils of coniferous forests this ratio is 27 or more [26].

The C:N ratio in horizon A, with the highest organic matter content, is 25.3 (Table 1). Microbial activity is facilitated by the good air permeability characteristic of rusty soil. Intensive decomposition and mineralization of a significant amount of organic matter present on the surface occurs, accompanied by the release of nutrients. The presented facts lead to the conclusion that this is a mesotrophic variety of rusty soil [26].

The granulometric composition of the soil is described as loose, slightly clayey sand, underlain by loose sand (Table 2). Such light sandy soils do not hinder the penetration of taproots, diagonal roots, or lateral roots of silver fir. They offer good aeration but are limited in their ability to retain moisture and nutrients [26].

**Table 2:** Granulometric composition. Percentage [%] of fractions passing through sieves of a given size.

Horizons	Sieve size [mm]								
	8	4	2	1	0.5	0.25	0.125	0.063	x < 0.063
A	0.00	0.00	1.25	1.45	6.98	31.59	28.79	25.40	4.54
Bv	0.00	0.00	0.97	2.80	14.77	45.10	25.59	9.53	1.24
BvC	0.00	1.87	1.58	1.78	4.41	30.94	33.79	21.50	4.13
C	0.00	0.00	0.00	0.80	4.08	55.71	35.80	3.05	0.57

#### Groundwater and forest management conditions.

The groundwater level, measured every ten days in a 3 m deep well located directly under the crowns of the fir trees, was below 3 m during the growing season (May–September) in 2024–2025. It should be noted that the same its position was recorded during the period of the most active underground runoff of melt water, replenishing groundwater reserves – in early spring, as well as during the autumn rains.

Survey area is situated within the IV Economic Activity Zone of the UNESCO World Heritage Site, where limited forestry operations are permitted [27]. These activities include the periodic felling of standing dead trees near forest roads, which are cut into logs of arbitrary length but not removed from the site. These logs, along with wind-felled or broken trees, now lie scattered across the area, often forming dense, nearly impassable accumulations. The volume of deadwood across an area of 1.4 hectares currently exceeds 700 m^3^ (Fig 1).

#### Study population spatial arrangement.

The population examined in this study is a fragment of an artificially established silver fir stand. It comprises 30 maternal specimens arranged in a linear “chain” formation, either as isolated individuals or in small clusters of 2–5 trees. This structure is accompanied by a diverse, multi-aged regeneration cohort of *A. alba*, all located within a fenced enclosure originally constructed in 1995.

#### Origin and silvicultural characteristics of trees, field research plan.

This fragment of the stand originated on the site of a former clear-cut. According to our research, the silver firs present in 2025 are 97 years old, having established from seed sowing of unknown origin that occurred in 1928.

A detailed root system study was conducted in a group of trees numbered 1–5 (Table 3, Fig 4). We selected these five trees because they form a small group of fir stands, in which the regeneration of *A. alba* is abundant under the crowns of the maternal trees, while secondary successional species are comparatively rare. It should be noted that group placement is most typical for silver fir [28]. The average distance between trees is 3.74 m, varying from 1.34 to 8.03 m [18]. That is, in this fragment of the forest stand, the microenvironment of the silver fir forest has been preserved to the greatest extent, and here, as we believe, the changes associated with the decay of the spruce component, including the allometry of the root system of the species under study, will be most representatively presented. The tallest silver fir, designated tree No.1, was chosen as a reference point for root length measurements.

**Table 3:** Sample collection points and their distance [m] from research points to silver fir tree No. 1.

**P**_**1**_	**P**_**2**_	**P**_**3**_	**P**_**4**_	**P**_**5**_	**P**_**6**_	**P**_**7**_	**P**_**8**_	**P**_**9**_	**P**_**10**_	**P**_**11**_	**P**_**12**_	**P**_**13**_	**P**_**14**_
5.50	9.04	22.09	25.81	23.54	7.43	21.40	25.45	20.91	22.70	23.98	17.98	10.10	5.76
**P**_**15**_	**P**_**16**_	**P**_**17**_	**P**_**18**_	**P**_**19**_	**P**_**20**_	**P**_**21**_	**P**_**22**_	**P**_**23**_	**P**_**24**_	**P**_**25**_	**P**_**26**_	**P**_**27**_	**P**_**28**_
9.67	15.57	22.22	16.04	16.04	17.39	16.11	5.62	16.29	6.78	17.20	6.20	6.22	20.78
**P**_**29**_	**P**_**30**_	**P**_**31**_	**P**_**32**_	**P**_**33**_	**P**_**34**_	**P**_**35**_	**P**_**36**_	**P**_**37**_	**P**_**38**_	**P**_**39**_	**P**_**40**_	**P**_**41**_	
6.40	21.20	20.70	23.10	10.38	12.66	10.38	5.13	3.93	4.63	2.10	3.20	10.40	

**Fig 4:**
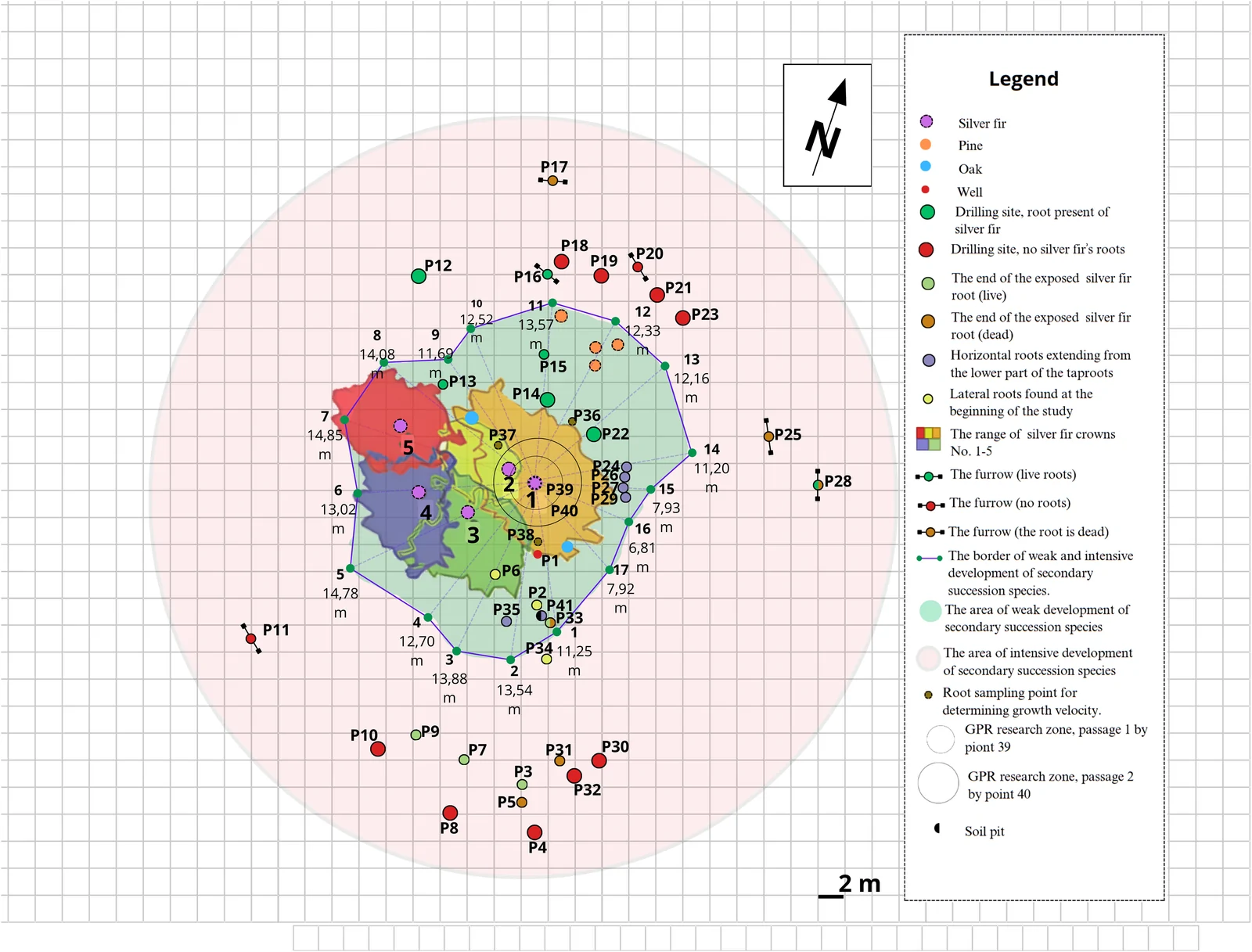
Scheme of conducting field research of the silver fir’s root system.

On the other hand, in the immediate vicinity of this biogroup (5.8 m), an oak tree over 140 years old remained, as well as a pine biogroup consisting of four trees (9.54 m) approximately 100 years old (Fig 4). These are the most common species in Polish forests, and when planning the study, we assumed that such microlocation of trees would allow us to assess the impact of interspecific root system competition. This circumstance also influenced our selection of these five trees for the study (Table 4). The zone free from interspecific competition between mature trees was located in the opposite direction, to the south-southwest, where spruce had previously grown.

**Table 4:** Characteristics of the studied silver fir trees.

No. of tree	Diameter [cm]			Height [m]			Crown length [m/%]	Trunk Vol.
	E-W	N-S	avg	1st dim	2nd dim	avg	in rel. to height	[m^3^]
1	69.0	68.8	68.9	34.5	34.6	34.6	26.0/75.1	5.74
2	24.9	25.9	25.4	24.9	25.9	25.4	18.1/71.3	0.69
3	48.6	49.0	48.8	29.0	29.7	29.4	22.7/77.2	2.61
4	34.0	33.3	33.5	25.1	25.2	25.2	17.9/71.0	1.13
5	36.5	35.0	35.8	22.1	22.6	22.4	16.4/73.2	1.17

#### Associated tree species and regeneration.

In addition to Scots pine, pedunculate oak, and silver fir, fragments of the original canopy layer, still preserved following the collapse of the stand, and scattered individuals across the site, as well as the undergrowth, host other forest-forming tree species typical of this habitat. All these species are listed in Table 5.

**Table 5:** Species of trees growing on the experimental plot.

Layer	Plant
Trees	Silver fir (*Abies alba* Mill.)
	Norway spruce (*Picea abies* (L.) H.Karst)
	Scots pine (*Pinus sylvestris* L.)
	European hornbeam (*Carpinus betulus* L.)
	Pedunculate oak (*Quercus robur* L.)
	Common birch (*Betula pendula* ROTH.)
	European mountain ash (*Sorbus aucuparia* L.)
Undergrowth	Silver fir (*Abies alba* Mill.)
	Norway spruce (*Picea abies* (L.) H.Karst)
	Scots pine (*Pinus sylvestris* L.)
	European hornbeam (*Carpinus betulus* L.)
	Pedunculate oak (*Quercus robur* L.)
	Common birch (*Betula pendula* ROTH.)
	European aspen (*Populus tremula* L.)
	Norway maple (*Acer platanoides* L.)
	Wild pear (*Pyrus communis* L.)

Despite the increase in lateral light availability following the removal of the *P. abies* canopy, the representation of undergrowth species, excluding *A. alba*, remains relatively low beneath the crowns of the biogroup of five silver fir trees.

In contrast, silver fir regeneration within this ecological niche is notably robust. In 2023, the density of *A. alba* seedlings reached 44,670 individuals per hectare in some plots. This figure aligns with regeneration levels observed in silver fir stands in the Carpathians, which are considered among the most successful and ecologically stable in Poland.

#### Diversity of shrub layer and secondary succession phytocoenoses.

This vegetation layer is characterized by a fairly significant species diversity, reflecting the growing conditions (Table 6).

**Table 6:** Species of bushes in the shrub layer.

Barcelona-nuts (*Corylus avellana* L.)
Raspberry (*Rubus idaeus* L.)
Ness Bramble (*Rubus nessensis* L.)
European red elder (*Sambucus racemosa* L.)
Whitethorn (*Crataegus monogyna* Jacq.)
English broom (*Cytisus scoparius* (L.) Link)

Following the elimination of Norway spruce in 2016, two primary types of secondary succession phytocoenoses emerged outside the under-crown zones of silver firs, replacing the herbaceous and cereal vegetation that had briefly dominated the open areas or became integrated with it: (I) Post-disturbance *R. idaeus* community (poor variant) This community is dominated by *R. idaeus*, with significant contributions from *R. nessensis* Hall, *Urtica dioica* L., and *Pteridium aquilinum* (L.) Kuhn. (II) Regenerating *Tilio-Carpinetum* community Characterized by the dominance of *C. betulus*, this community also includes *B. pendula*, *S. aucuparia*, and *C. avellana* L., reflecting a typical mesophytic deciduous forest structure (Table 6).

At present, a dynamic transition is underway, with the *R. idaeus* community gradually being replaced by the regenerating *Tilio-Carpinetum* assemblage.

### Methods and equipment used in the study

#### Ground penetrating radar.

Modern investigations of tree root systems increasingly utilize ground penetrating radar (GPR), a non-destructive technique that facilitates fieldwork by enabling repeated measurements at the same location. This makes GPR particularly effective for monitoring long-term changes. However, its application is generally limited to small-scale studies and root diameters larger than 1 cm, as it struggles to reliably detect thinner roots that play a crucial role in resource absorption [14,15,29–31].

For this study, the Zond-12e “advanced” ground-penetrating radar (GPR) system was used, equipped with shielded transmitting and receiving antennas operating at frequencies of 500 MHz and 900 MHz, with penetration depths of 5–10 m and 2–5 m, respectively. Data were acquired for a trace duration of 100 ns and 256 samples per trace. In the analysis, a relative permittivity of 10 was assumed, based on field conditions for sandy soil with low water content. The data were subjected to noise filtering and background correction. Selected GPR profile points were verified by root uncovering, confirming the consistency of the radar signals with the actual root structure. The study highlighted the possibility of ambiguous signals and potentially false positives in individual GPR passes, such as diffractions from root overlap or from shallow roots. The measurements were carried out using two concentric transects (Fig 4 P_39_, P_40_) around the fir trunk No. 1 and radial crossings as controls.

GPR performs optimally in dry, sandy soils due to minimal signal attenuation [32,33]. The mechanical composition of the soil at the study site, combined with a groundwater level below 3 meters, results in low soil moisture. These conditions are favorable for obtaining reliable data on the architecture of the thickest root structures, in particular the diagonal roots.

#### Traditional excavation method.

Although invasive and labor-intensive, traditional root excavation remains the most accurate method for studying vertical and lateral roots—especially those extending far from the tree trunk [30]. This is particularly important in upper soil horizons, where roots often intermingle with the underground structures of neighboring tree species. To date, no alternative approach offers a comparably objective assessment of root penetration parameters.

Once a silver fir root was located using the trench method, excavation was extended along its entire length until the terminal point was reached. Immediately after measurement, photographic documentation and sampling were performed, and then all was filled with soil.

The trench method involved constructing narrow furrows ranging from 0.5 to 4.5 meters in length and up to 25 cm in depth, oriented approximately perpendicular to the anticipated direction of lateral root growth (Fig 4 P_11_, P_17_, P_20_, etc.). However, quite often the silver fir root was discovered during random spot digging. When rapid species identification confirmed the root belonged to silver fir, further manipulations were similar to those described above.

#### Soil coring method.

A less labor-intensive, though still mechanically invasive, method applied in this study was soil coring using specialized augers [15]. While this technique facilitates root sampling, its effectiveness is constrained by the low likelihood of intercepting roots, especially those positioned far from the tree trunk. As a result, the representativeness of the data may be limited. Nonetheless, soil coring was used to complement other methodological approaches.

The primary tool used was the Eijkelkamp soil auger gouge, featuring a 30 cm-long open groove at the bottom, a diameter of 35 mm, and a sampling depth range of 0–30 cm, yielding a sample volume of 288.5 cm^3^.

Monoliths extracted with the auger were sieved using a 4 mm mesh. When fragments of silver fir roots were identified among the retained material, the distance from the sampling point to Tree No. 1 was recorded. To investigate the presence of absorption roots at greater depths, two soil profiles were excavated to depths of 160–170 cm, located 6.40 m and 10.38 m from Tree No. 1 (Fig 4, Points P_24_, P_26_, P_27_, P_29_, P_35_ These profiles were further deeped by an additional 1 meter using drilling techniques.

#### Field identification of *A. alba* roots.

Based on field observations, several distinctive visual characteristics were identified for thick lateral roots (diameter > 1 cm), which allow them to be distinguished from oak and pine roots growing near tree groups No. 1–5.

(I) Surface texture and coloration. The bark is slightly rough to the touch and has a characteristic carmine-red hue, visible under narrow strips of peeling periderm. (II) Bulges. “Chains” of protruding structures are visible on the root surface, increasing in size with growth root diameter. These structures are arranged parallel and surround the root in radial cross-section. (III) Morphological flattening. The distinct ellipsoidal shape in the radial plane of the root further facilitates visual differentiation (Fig 5a.

**Fig 5:**
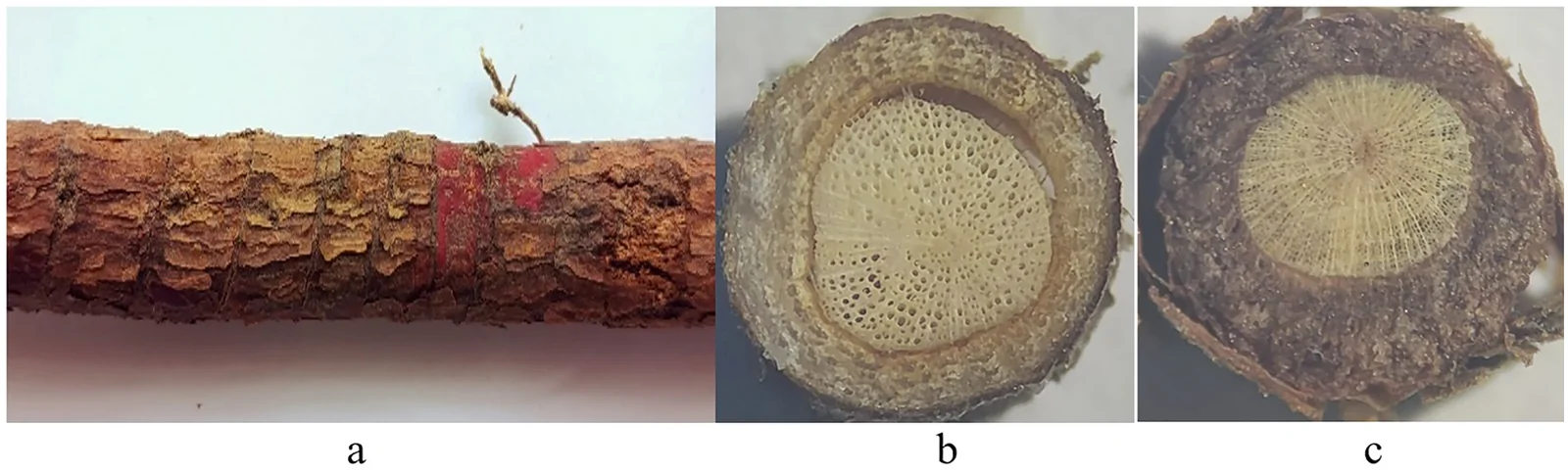
a. Peeling periderm, revealing red-carmine bark. Chains of bulges on the surface of thick roots (photo by K. Wilamowski); Images of cross-sections of thin roots 24 hours after their preparation, obtained in the field: b. hornbeam, c. silver fir (photos by A. Marozau).

Visual identification of fine lateral roots (diameter 2–3 mm) of silver fir in the field is more difficult than recognizing thick ones. This is due to their significant visual similarity to the fine roots of hornbeam (*Carpinus betulus* L.), which grew beyond the undercrown zone of tree group No. 1–5 during secondary succession. Fine silver fir roots are also found here. Diagnostic characteristics effective for thick roots are inefficient in this case; however, using a number of new criteria, it was possible to differentiate silver fir and hornbeam roots.

(I) Structure of the vascular system. Hornbeam roots have large and small vessels, clearly visible in cross-sections as evenly distributed openings, which are absent in silver fir. (II) Bark thickness. Hornbeam bark is much thinner than silver fir one. (III) Expression of growth rings. By hornbeam, the boundaries between early and late wood, as well as the annual growth rings, are virtually indistinguishable, whereas in silver fir they are more clearly differentiated. The cross-section of the silver fir bark has more dark brown-red hue. (IV) Medullary rays. They are more visible in root sections of silver fir than in hornbeam. (V) Coloration. Fresh cross-sections of silver fir roots have light carmine hue, while hornbeam roots appear almost white. (VI) Cross-sectional shape. Silver fir roots have an oval cross-section (which is also observed in thick roots), whereas by hornbeam roots they are invariably round. Additionally (VII), a physicomechanical characteristic – wood hardness. When cut, the wood of silver fir roots is noticeably softer than that of hornbeam, which is sometimes called “ironwood”. The given characteristics allow to confidently identifing lateral roots of *A. alba* of different thicknesses directly in situ in mixed stands (Fig 5b, 5c).

#### Microscopic analysis of root tips.

Microscopic analysis was used to determine the species identity of the finest tips of lateral roots and deep absorptive roots of silver fir with a diameter of less than 1 mm (Fig 6).

**Fig 6:**
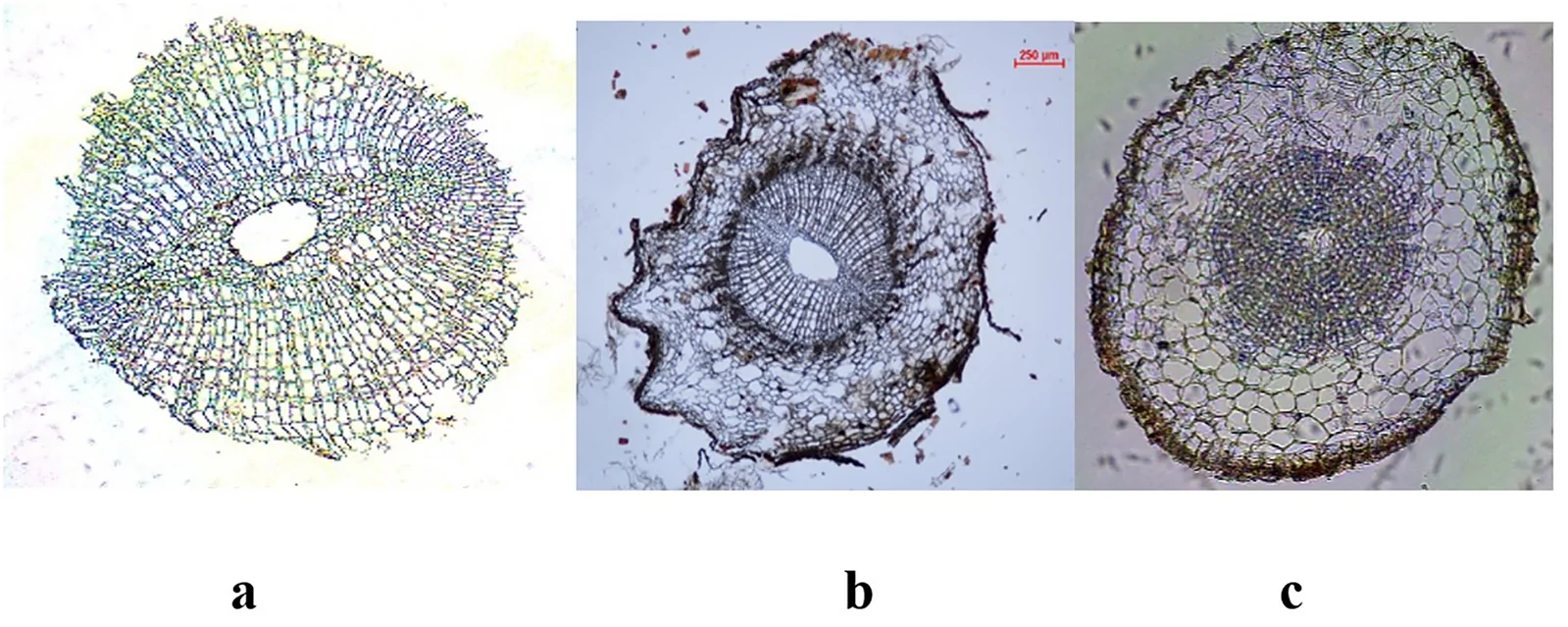
a. Radial cross-section of the end of a lateral silver fir root at a distance of 22.09 m from tree 1 (P_3_); Fine absorptive roots extending from the skeletal roots at a distance (L) and depth (G): b. L = 6.40–6.78 m, G = 1.95–2.20 m; c. L = 10.38 m, G = 2.15–2.40 m (photos by E. Bagińska, A. Marozau).

This was essential for accurately assessing their maximum length, as the visual diagnostic features previously described are not applicable at this scale. Microscopy was also used to count annual growth rings in thicker roots (diameter 0.8–1.3 cm), enabling estimation of growth rates under varying below-ground competition conditions.

Microscopic preparations were prepared using an OPTA-TECH MR-315 rotary histopathological microtome, following the xylem anatomical analysis protocol outlined by Arx et al. [34]. Imaging was performed with an OPTA-TECH microscope equipped with an MI6 OPTA-TECH camera and Capture V 2.0 software.

## Results

### Penetration depth of fir roots

The study, conducted using ground-penetrating radar with measurements taken along circular paths at distances 2.1 m and 3.2 m from the trunk of fir No. 1 (Fig 4, points P_39_, P_40_), allowed the detection of fir root system structures within a depth range of approximately 1.0 m to 2.5 m. The results showed a dense network of surface roots belonging to the silver fir regeneration, abundantly represented under the crowns of old trees (line at a depth of 0 m). Below, at a depth of about 1 m, an irregular broken line of lateral fir roots can be observed, and at a depth of 1–2.5 m, discontinuous diffractions from diagonal roots can be seen (Fig 7), which is consistent with the measurement of roots in the excavation. Recordings indicating depths greater than 2.5 m most likely represent measurement errors caused by diagonal roots protruding from the ground surface near the trunk. Excavation of the taproot revealed that its penetration depth into the ground is approximately the same as that of the diagonal roots about 2.5 meters.

**Fig 7:**
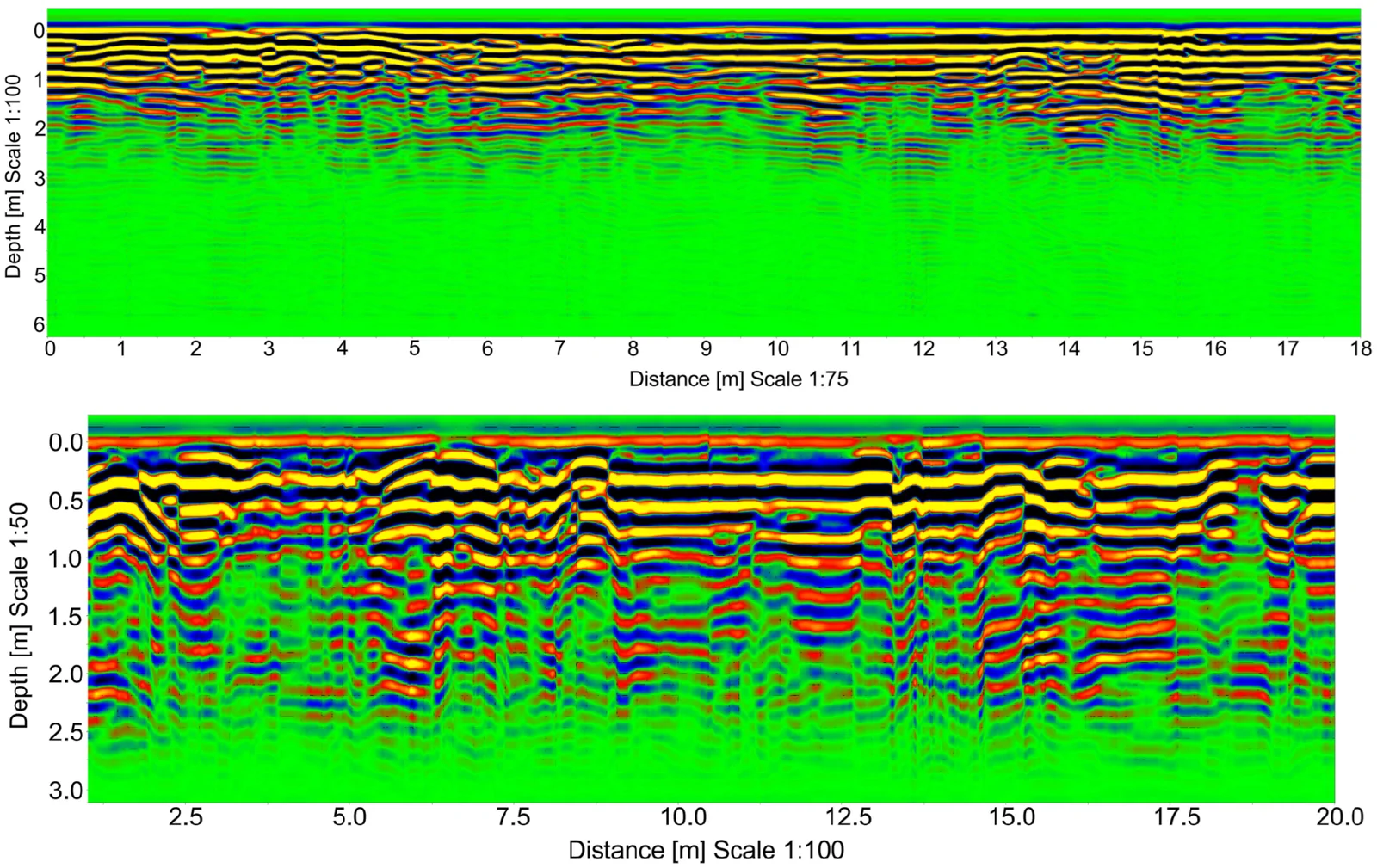
(Top) 500 MHz radargram for the depth of diagonal roots obtained as a result of the first pass (length = 18 m) around tree No. 1. (Bottom) 900 MHz radargram for the depth of diagonal roots obtained as a result of the second pass (length = 20 m) around tree No. 1 (photos by K. Zabielska-Adamska, P. Dobrzycki).

Thus, visualization of subsurface soil layers using ground-penetrating radar (Fig 7) revealed no differences in the depth of diagonal silver fir roots in Białowieża Forest compared with literature data. Excavation of the vertical taproot also revealed no differences in its length from known data. This served as the basis for rejecting hypothesis I (the “Introduction” section).

### Spread of lateral roots

Lateral roots of silver fir were identified during soil studies. Initial spontaneous detection occurred at distances of 9.04 m, 7.43 m and 12.66 m, corresponding to points P_2_, P_6_ and P_34_, respectively (Fig 4).

A point P_34_ the most distant from tree No. 1 at that time, roots with diameters ranging from approximately 20.0 to 35.0 mm were found at relatively shallow depths (20–50 cm), indirectly indicated their considerable length.

Thus, analysis of the initial field data revealed that existing literature likely underestimates the maximum lateral root length of silver fir [11,13]. In 2024–2025, terminal points of silver fir lateral roots were discovered at maximal distances from tree No. 1 within competition-free zones with old trees, specifically at 22.09 m, 21.4 m, and 20.91 m respectively (Table 3, Fig 4).

In the area where dead spruce trees were located, intensive development of the secondary successional phytocenosis *Tilio-Carpinetum* began. Competition with the numerous representatives of this new plant community may be even stronger and involve more participants than competition observed with their above-ground organs [35]. The image of intertwined roots from different species shows that the *Tilio-Carpinetum* community exerts a suppressive effect on the terminal ends of silver fir lateral roots, even causing their mortality (Fig 8(a,b)).

**Fig 8:**
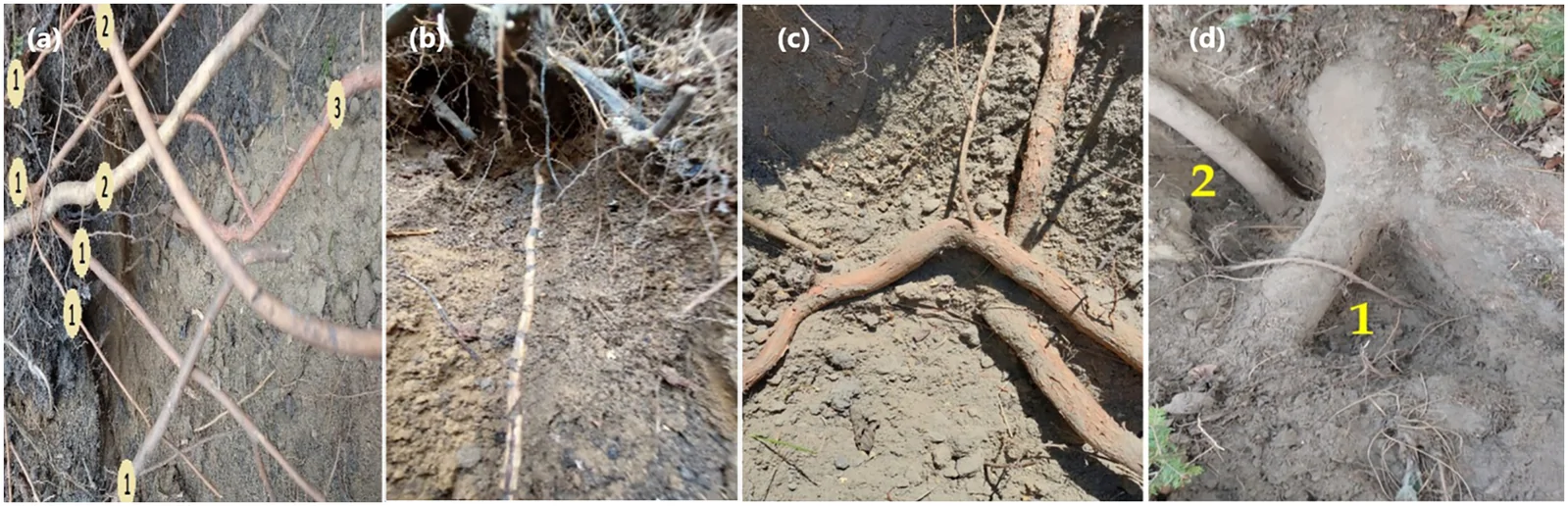
(a) Network of roots of (1) *C. betulus* and (2) *S. aucuparia* competes with the roots of (3) *A. alba* in the humus horizon; (b) A dead lateral root of *A. alba*, 23.54 m long (P_5_), influenced by the root systems of secondary successional species (top); (c) The lateral roots of *A. alba* avoid competition; (d) Overcoming mechanical obstacles with lateral roots of silver fir (photo A. Marozau).

This is further evidenced by observations from certain parts of the active development zone of this phytocenosis, for example at point P_3_, where the longest living lateral root of *A. alba* was recorded. In this area, the numbers of pioneer species are notably high, with *C. betulus* reaching 777 individuals per hectare and *S. aucuparia* 888 individuals per hectare, growing up to 4–7 meters in height.

In order to avoid competition from the root systems of representatives of the secondary succession phytocenosis, the lateral roots of silver fir sometimes display notable plasticity, altering their typical horizontal rectilinear growth direction to an almost perpendicular orientation (Fig 8(c)). Otherwise, the outcome of such competition could be detrimental for *A. alba* (Fig 8(b)).

When encountering mechanical obstacles (in this case, a thick diagonal root) (Fig 8(d), the lateral roots of the silver fir can either “jump” over them (1) or “crawl” under them (2). This again convincingly demonstrates their pronounced ability to change the direction of growth if necessary. In this case, already in the vertical plane.

In the case where there are no mechanical obstacles of any kind, a powerful compact “stream” is formed, consisting of several thick lateral roots, approximately the same thickness (Fig 9(a)) or, on the contrary, significantly different in diameter at a depth of 0.2–1.0 m (Fig 9(b)), purposefully moving into zones of no underground competition of old oak and pine trees.

**Fig 9:**
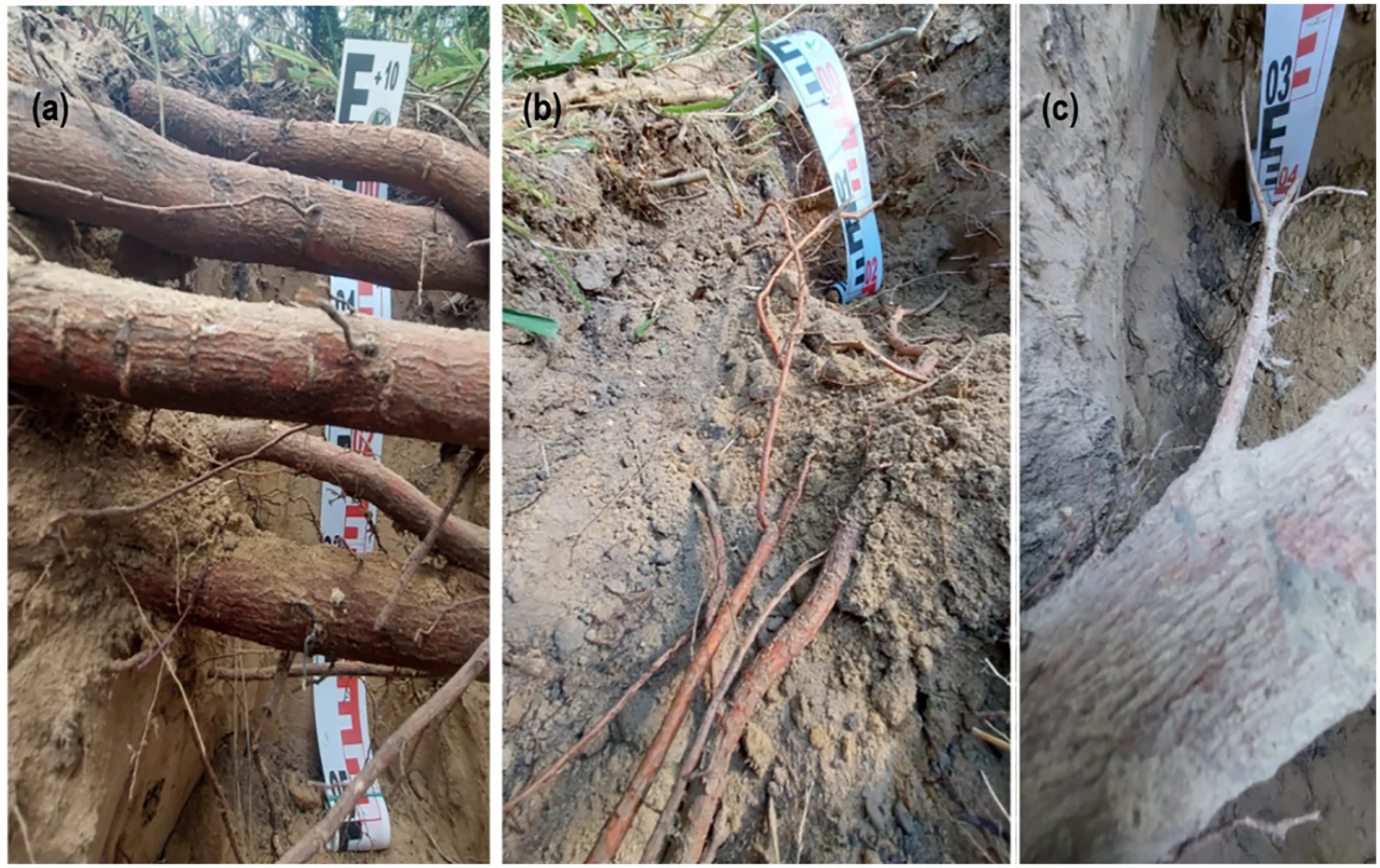
(a)(b) Silver fir’s roots traffic; (c) Anchor root of silver fir’s lateral root (photo A. Marozau).

On the lateral roots, especially on their thicker segments, there are vertical anchor roots “trying to find” groundwater (Fig 9(c)). Their important role is to provide greater mechanical stability to the massive fir trees, which are now completely exposed to the winds.

Due to the decomposition of dead wood the upper part of the soil profile became more eutrophicated (Fig 10(a)). In this situation, the fine lateral roots of the silver fir begin to emerge into the more fertile humus level A: 4–18 cm unless, of course, they not encounter more and more fierce competition from secondary succession species, as mentioned above.

**Fig 10:**
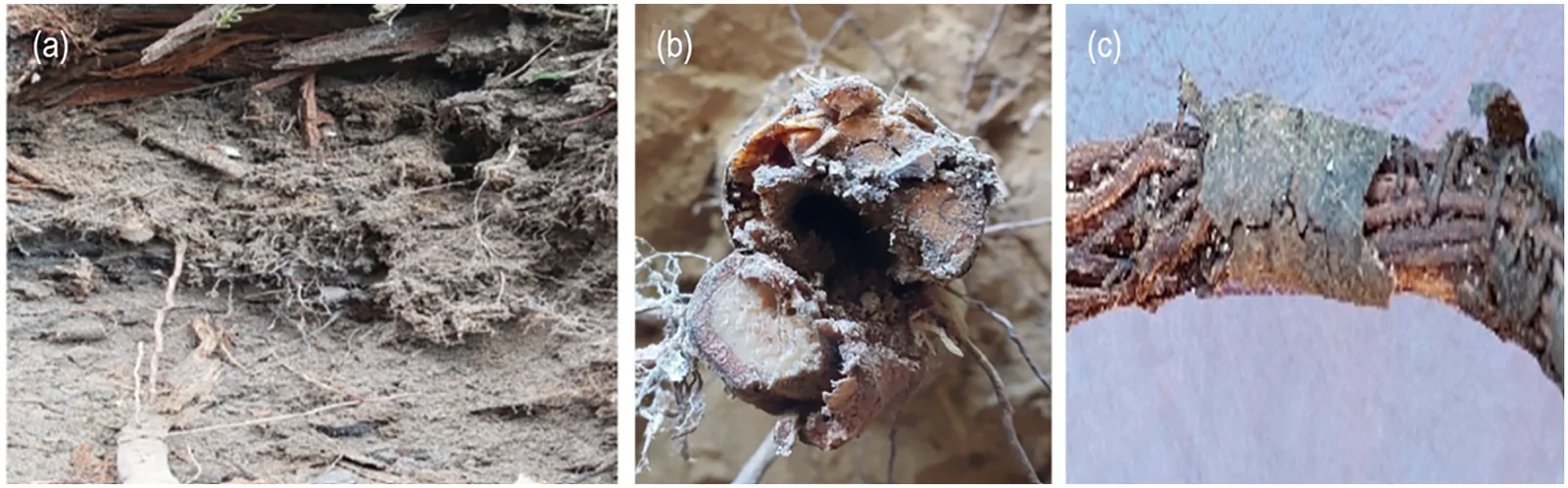
(a) The association of silver fir roots with dead tree decomposition microlocations; (b) The dead root of a spruce (top), which died in 2012–2016, is used by a silver fir (bottom); (c) A bunch of thin lateral roots of silver fir in a cocoon of decomposed spruce root (photo A. Marozau).

Silver fir lateral roots advantageously utilize dead spruce roots of various thicknesses as dispersal pathways and as nutrient sources through mycorrhizal associations [36,37] (Fig 10 (b, c)). Similar interactions have been documented for *Pseudotsuga* *menziesii* by [35], where fine roots were absent in mineral soil but present in buried dead wood.

In the absence of competition from the root systems of old pine and oak trees (Fig 4, significantly faster lateral root growth of silver fir was recorded compared to conditions with competition. For example, a lateral root reached a distance of 10.38 m in 10 years (Fig 11(a)), indicating an average annual growth rate of 1 m. In contrast, it took 12 years (Fig 11(b)) to cover half that distance (5.13 m) in the direction of a pine biogroup, corresponding to an annual growth rate of approximately 0.4 m, representing a slowdown by a factor of 2.5.

**Fig 11:**
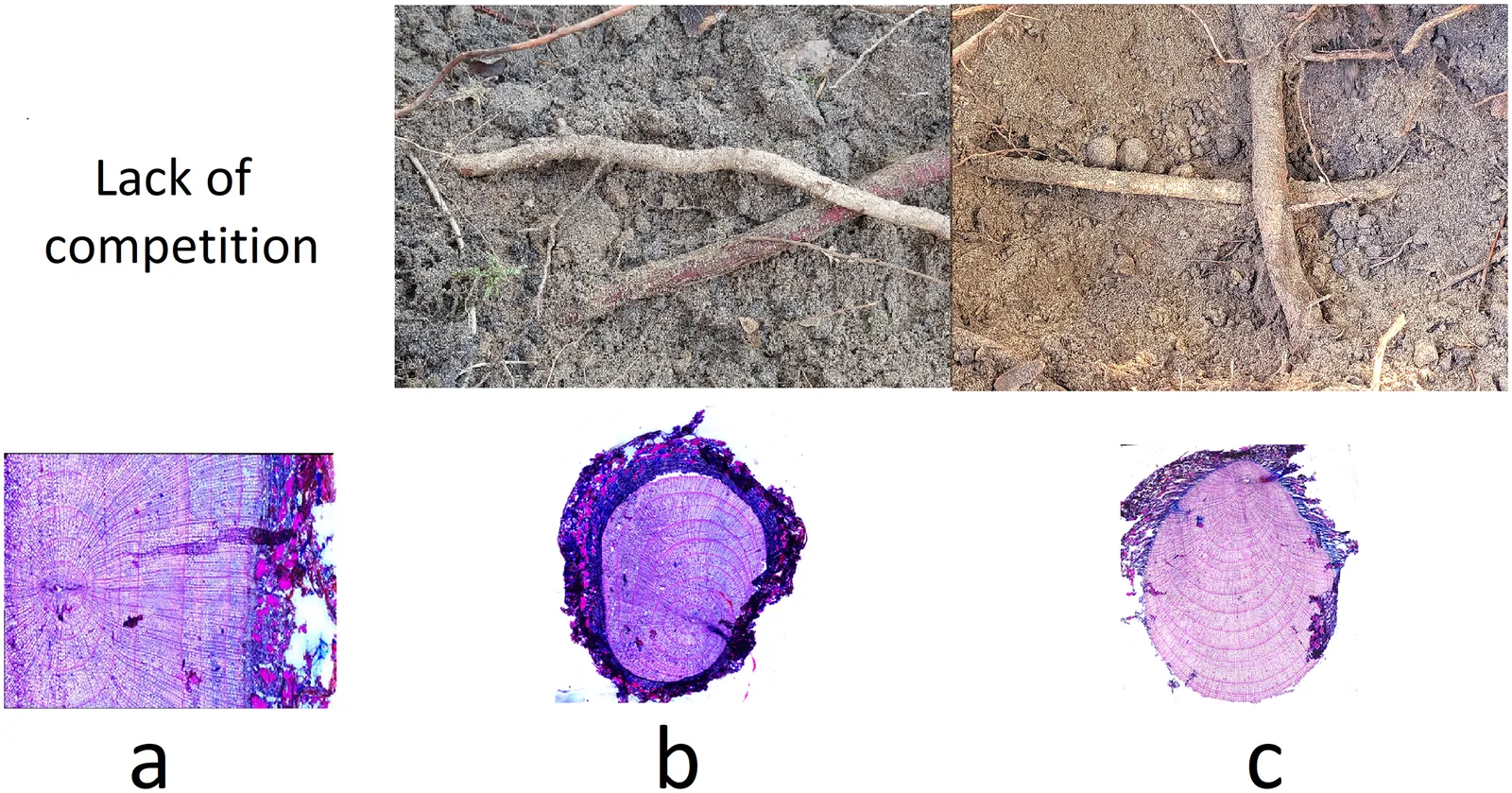
The growth rate of lateral roots of silver fir depending on the conditions of competition: a. there is no competition, b. competition with pine roots, c. competition with oak roots (photos E. Bagińska, A. Marozau).

The most pronounced growth reduction occurred under competition with oak roots, where a silver fir lateral root grew to 3.93 m in approximately 25 years (Fig 11(c)), yielding an average annual growth of only 0.16 m. This reflects a decrease by a factor of 6.5 compared to the no-competition scenario and almost threefold compared to competition with pine.

### Spread of deep absorptive roots

Methodological challenges related to studying deep, fine lateral roots extending from taproot and diagonal roots contribute to the limited literature on this topic and its limited representation in this study. However, the recorded points of their discovery (Fig 6b, 6c), allow us to assume that the area they occupy is possibly comparable to the area of the under-crown space of a biogroup of five trees [38], at a depth of about 2.5 m.

## Discussion

According to Boratyński [12], in wet habitats and on shallow rocky soils, silver fir develops a flat (“plate-shaped”) root system, although the dimensions of this system are not specified by the author. At the same time, as emphasized by Gulder [13], even under severe waterlogging conditions, the taproot-type root system characteristic of *A. alba* remains unchanged, although it may not be prominently expressed [39]. Similarly, Kutschera and Lichtenegger [11] reported a constant root system morphology for studied species.

This morphological conservatism is somewhat unexpected, given that many species with broad ecological amplitudes, such as *Pinus sylvestris* [40], known as a double xerophyte [41], exhibit considerable plasticity in adapting their root systems to varying environmental conditions. For example, in response to soil moisture deficits (physical dryness), pine develops a deep root system, whereas in conditions of excess moisture (physiological dryness), it forms a superficial root system [42–44].

Tree phenotype, including the root system, is shaped not only by genetic factors but also by external influences such as soil type, hydrological regime, soil granulometric structure, nutritional conditions, mechanical barriers, and competitive interactions [45].

In existing studies on *A. alba* known to us, the impact of competitive relationships has not been prominently reflected [11–15]. Our study, however, found that silver fir lateral roots have significant growth potential but are highly sensitive to competition, particularly from roots of old oak trees and from numerous individuals of the secondary succession phytocenosis *Tilio-Carpinetum*.

Regarding other external factors, the foremost is the granulometric structure of the loose, slightly clayey, well-drained sandy soil characteristic of the study area. This substrate does not hinder horizontal root propagation and, importantly, also does not impede vertical root growth [46]. Since the research site is fenced, mechanical soil compaction by wild animals, such as bison males weighing up to 700 kg, can be excluded.

A second, more complex and arguably key environmental factor influencing root phenotype is moisture availability, which comprises several interconnected components.

Firstly, at the end of the last century, a marked trend of groundwater level decline was observed across all habitat types in the Forest, including mesotrophic deciduous forests [2,24]. Groundwater levels dropped below 3 m in some years, and recent assessments suggest further deterioration [23].

As noted in the Introduction, the taproot system of *A. alba* generally reaches depths of 1.5–2 m, exceptionally up to 3 m [11,13,14]. Our research indicates that skeletal orthotropic root extends to about 2.5 m. Diagonal roots reach approximately the same depth. Given that mechanical fractions of the parent rock (horizon C) are predominantly medium (55.7%) and fine (35.8%) sand (Table 2), the height of the capillary fringe can be only a few tens of centimeters [47]. Therefore, groundwater is effectively inaccessible to silver fir roots. Above the capillary fringe, other soil moisture forms, gravitational and vaporous, are present only in minimal amounts, owing to the low water-holding capacity and high permeability dictated by granulometry.

At the same time, under drought conditions, the silver fir root system may be susceptible to embolism; according to Nourtier et al. [48], the deepest roots are particularly vulnerable to this phenomenon.

Secondly, due to the inability to access groundwater, silver fir exhibited a need to diversify its sources of water and mineral nutrition. This led to an increased reliance on atmospheric precipitation, including horizontal precipitation forms such as dew, fog, icing, and frost. The contribution of these forms to total annual precipitation can reach 9.4% [49,50], and according to some sources, may be as high as 30% [51]. Their importance is greater in mountainous regions, at forest edges, and on open surfaces [52]. Indeed, such an open area surrounded the silver fir biogroup in the initial years following the collapse of the spruce stand.

It was found that about 45% of the precipitation gets lost as interception loss from this dense forest canopy [53]. At the same time, silver fir is characterized by the most pronounced interception among local species [54]. Consequently, the extension of lateral roots beyond the crowns and the resulting increase in moisture capture area were stimulated, among other factors, by a distinct bipolarity of microclimatic conditions—differences under the crowns and open environments space.

Further facilitation of root system phenotypic modification is attributed to the active decomposition of dead spruce wood concentrated beyond the silver fir under-crown space, including decomposing roots, which releases significant amounts of CO_2_. Elevated CO_2_ concentrations have been shown to stimulate fine lateral root growth, which plays a major role in water and nutrient uptake; for example, in poplars, fine root growth can increase by 25–45% under elevated CO_2_ [55]. Free from carbon starvation and mineral nutrient deficiencies, lateral roots readily penetrate loose soil. This phenomenon increases the exploitation area of hydromineral resources in the upper soil horizons, ensuring normal silver fir physiological functioning.

Therefore, analysis of literature and experimental data leads to the conclusion that the limited penetration depth of the taproot and diagonal roots (approximately 2.5 m) precludes reaching the capillary fringe of markedly lowered groundwater. However, the notably extended lateral roots, reaching up to 22.09 m and surpassing all previously reported values, enable *A. alba* to not only persist but also maintain high vitality and performance. Thus, working hypothesis II (see Introduction) was confirmed.

Consequently, in the dilemma faced by silver fir regarding survival strategies—whether to enhance taproot and diagonal roots growth or to activate horizontal lateral roots—the latter clearly prevails as the preferable and more feasible option, but only if there is no competition from secondary successional species and other mature trees (Fig 8, 11).

No data were found in the literature regarding the spatial distribution parameters of absorptive roots extending from the taproot and diagonal roots. Therefore, lacking comparative analyses, we can only confirm that the area occupied by these roots is substantial and likely comparable to the horizontal projection area of crowns trees No. 1–5 [38].

In the absence of competition, lateral root growth and development in old-growth silver fir under challenging environmental conditions give rise to a synergistic phenomenon that communicates between above-ground and below-ground organs, producing reciprocal positive effects. Specifically, increased spread of horizontal roots positively influences crown and trunk growth and development (Table 4), leading to improved fruiting and favoring natural generative reproduction. Conversely, a well-developed above-ground structure stimulates root growth. Ultimately, each individual tree, as the fundamental structural unit of a forest stand—the principal component of the forest ecosystem—becomes more resilient to environmental stresses.

Given the ecological tolerance of *A. alba* beyond its natural range in northeastern Poland [38] and its considerable longevity, the presence of this late-successional species should be considered a valuable asset for successful restoration of forest ecosystems. A management strategy emphasizing long-term preservation of the “silver fir skeleton” within the forest ecosystem, even after the elimination of other components, is advisable in light of increasing challenges related to the declining stability of forest-forming species under climate change [56]. When planning conservation measures, the two-way feedback between vegetation and climate should be taken into account [57].

The findings presented here also contribute to understanding the adaptive capacity of silver fir under ongoing climate change. The observed expansion of lateral roots suggests that mature trees may compensate for reduced access to groundwater through increased exploitation of near-surface soil resources. Such plastic responses may play an important role in maintaining forest resilience under future drought scenarios and changing stand structures.

## Conclusion

The traditional concept of the silver fir root system as a typical taproot with four to five powerful diagonal roots should be expanded to include the capacity for dynamic modification under specific conditions, such as absence of below-ground competition and low groundwater levels. This plasticity occurs even in relatively old silver fir trees, approximately 100 years of age and forms the basis for adaptation to complex environmental stressors.

A more efficient spatial root architecture develops, notably a branched system of lateral roots spreading horizontally within soil depths of 0.2 to 1.0 meters, extending laterally up to 22.09 meters. This configuration satisfies water and nutrient demands and increases mechanical stability, which is particularly important as silver firs are increasingly exposed to strong winds following spruce canopy loss.

Within the same silver fir individual, the growth rate of lateral roots in directions of competitors (pine, oak) slows down significantly and simultaneously accelerates in the direction free from competition.

The depth of the taproot and diagonal roots is approximately 2.5 m, insufficient to access groundwater. Fine absorptive roots arise from these at depths to 1.95–2.40 m, extending considerably up to 6.40 to 10.38 m, with an absorptive surface potentially comparable to the silver fir under-crown space.

Several limitations of the present study should be acknowledged. The investigation was conducted at a single site and focused on a relatively small number of mature trees. Consequently, the results should be interpreted cautiously when extrapolating to other ecological conditions or populations of *Abies alba*. Furthermore, despite the use of complementary methods, detailed quantification of the finest root fractions remains challenging.
